# A nomogram for risk prediction in patients with heart failure and diabetes: Development and validation

**DOI:** 10.1097/MD.0000000000049341

**Published:** 2026-06-19

**Authors:** Zhe Zhang, Dengao Li, Jumin Zhao, Huiting Ma, Fei Wang, Qinglian Hao

**Affiliations:** a College of Integrated Circuits, Taiyuan University of Technology, Taiyuan, China; b College of Computer Science and Technology (College of Data Science), Taiyuan University of Technology, Taiyuan, China; c Department of Cardiovascular Medicine, Shanxi Cardiovascular Hospital, Taiyuan, China; d Department of Cardiovascular Medicine, Shanxi Provincial Integrated TCM and WM Hospital, Taiyuan , China.

**Keywords:** heart failure, LASSO, logistic regression, nomogram, NT-proBNP, risk prediction, screening, type 2 diabetes mellitus

## Abstract

Heart failure (HF) commonly coexists with type 2 diabetes mellitus (T2DM), and this combination is linked to a heavier symptom burden and less favorable clinical outcomes. In this retrospective single-center study, a total of 958 consecutive patients were included, among whom 453 had T2DM, with a mean age of 68.23 ± 5.76 years. The diagnosis of HF was confirmed by a multidisciplinary team in accordance with the European Society of Cardiology criteria, and 232 patients were found to have both T2DM and HF. Relative to diabetic patients without HF, those with HF more often presented with dyspnea or fatigue, paroxysmal nocturnal dyspnea/orthopnea, and ankle swelling or nocturia (all *P* < .001). They also showed higher rates of wheezing/rhonchi (*P* = .021), fluid and sodium retention (*P* = .008), ST–T abnormalities (*P* = .033), abnormal Q-waves (*P* = .001), and NT-proBNP levels ≥ 15 pmol/L (approximately 125 pg/mL; *P* < .001). Potential predictors were first selected using least absolute shrinkage and selection operator (LASSO) regression, after which multivariable logistic regression was performed to construct a nomogram for predicting HF risk in patients with T2DM. The multivariable model indicated that ST–T abnormalities, NT-proBNP, ischemic heart disease, and atrial fibrillation were independently related to HF (all *P* < .05). The nomogram exhibited strong apparent discriminatory ability together with satisfactory calibration, suggesting that NT-proBNP-based risk stratification may be useful for HF screening in individuals with T2DM.

## 1. Introduction

Heart failure is a population health issue and a public health concern that endangers people’s health.^[1,2]^ According to statistics, between 1% and 2% of people in affluent nations have heart failure (HF). The illness also has a high hospitalization rate and high financial cost, which puts a significant socioeconomic strain on society.^[1,2]^ Heart failure and type 2 diabetes mellitus are becoming more common conditions, and they are linked to higher rates of morbidity and death.^[3]^ It has been estimated that up to 22% of persons with type 2 diabetes in the United States also have heart failure.^[4]^ Glycemic irregularities have a role in the increased risk of heart failure seen in people with type 2 diabetes mellitus.^[5]^

Heart failure is again one of the most frequent consequences of cardiovascular disease, which is increased by type 2 diabetes.^[6]^ Heart failure is again one of the most frequent consequences of cardiovascular disease, which is increased by type 2 diabetes.^[7]^ More than 90% of cases of diabetes are type 2, which is defined by insulin resistance.^[8]^ Heart failure is twice as common in people with type 2 diabetes as it is in those without the disease.^[9]^ Diabetes mellitus contributes to the development of heart failure primarily through direct impairment of cardiac function and indirectly through hypertension, renal insufficiency, obesity, and other metabolic diseases.^[8]^ Unlike myocardial infarction, the risk of heart failure in type 2 diabetes exists even in people with optimal control of traditional risk factors such as smoking, hyperlipidemia, blood glucose levels, and hypertension. If a patient has 2 comorbidities, their prognosis is poorer, thus treating and preventing both issues is crucial.^[10]^

Unlike myocardial infarction, the risk of heart failure in type 2 diabetes exists even in people with optimal control of traditional risk factors such as smoking, hyperlipidemia, blood glucose levels, and hypertension. If a patient has 2 comorbidities, their prognosis is poorer, thus treating and preventing both issues is crucial.

## 2. Materials and methods

This study was approved by the Ethics Committee of Taiyuan University of Technology. This study is a single-center retrospective study. This study was approved by the ethics committee of our hospital and the institutional review board (IRB). All patients were informed and signed an informed consent form.

### 2.1. Study population

All patients included in this study were consecutively recruited between January 1, 2020 and February 1, 2024. Inclusion criteria were: age ≥ 18 years; diagnosis of type 2 diabetes mellitus (T2DM) according to established clinical criteria; and availability of complete clinical, laboratory, and echocardiographic data. Exclusion criteria included: type 1 diabetes mellitus; uncontrolled diabetic complications (e.g., proliferative retinopathy); recent use of GLP-1 (glucagon-like peptide-1) receptor agonists (within 90 days); and significant weight change (>5 kg) prior to enrollment.^[11]^ Patients were excluded if their clinical records were incomplete or if key data required for model construction were missing, including demographic characteristics, clinical history, laboratory indicators, electrocardiographic findings, echocardiographic parameters, or outcome assessment. Therefore, only patients with complete clinical information were included in the final analysis.

### 2.2. Outcome

Samples of blood were drawn and examined. NT-proBNP serum concentrations were determined using an immunoradiometric assay that is noncompetitive (Roche Inc., Mannheim, Germany). At last, GE Vingmed Ultrasound AS, Horten, Norway’s cardiac sonographers with training and expertise used a General Electric Vivid 7 imaging system equipment to do echocardiogram. Every participant’s echocardiographic data were evaluated by a skilled cardiologist. Doppler color analysis, mitral annular septal and lateral wall Doppler tissue imaging (DTI), *M*-mode parameters, and 2D transthoracic echocardiography parameters were all employed. Early diastolic mitral flow velocity (*E*), atrial contraction (*A*) wave velocity, and *E* deceleration time were all determined from the mitral inflow profile. *E*/*e*′12 is calculated by measuring the early diastolic mitral annular wall velocity, or *e*′ velocity. Determine the *E*/*A*, or early atrial LV filling ratio. Determine the ratio of systolic to diastolic forward blood flow (S/D) by measuring the flow velocity in the left or right upper pulmonary vein. LVH2 was the classification given to women whose left ventricular mass index (LVMi) was >95 g/m and >115 g/m^2^ in males. LVMi was calculated using the *M*-mode (Devereux and Reichek’s formula).

A panel consisting of 2 cardiologists and 1 general practitioner determined whether or not heart failure (HF) existed, according to the European Society of Cardiology’s standards on the subject.

### 2.3. Prognostic nomogram analysis

LASSO (least absolute shrinkage and selection operator) was used to reduce multivariate data and choose risk factors for diabetics with heart failure. In the training set, nonzero LASSO regression coefficients were used. Multiple logistic regression analysis was performed on selected variables in the LASSO regression model in order to construct a prediction model. A calibration curve was created in order to assess the nonadhesive coating nomogram’s calibration, and the Harrell concordance index was computed in order to measure the nomogram’s discriminative power. To evaluate the net benefit and determine if a decision curve analysis is necessary to identify noncompliance nomograms that are clinically significant. The appropriate ROC curve (AUC) 13 from the training and validation toolbox was used to assess the clinical factor model’s diagnostic efficacy.^[12]^

### 2.4. Statistical analysis

SPSS software (IBM Corp.) was used for conducting the statistical analysis. In univariate analysis, the Mann–Whitney *U* test was used to compare groups, and the Pearson correlation was employed to assess the linear connection between 2 continuous variables. To compare proportions, Fisher exact test was used. A *P*-value of <.05 was considered noteworthy.

## 3. Results

### 3.1. Baseline characteristic

In all, 958 patients were involved in this investigation. A total of 265 male patients and 453 patients with a mean age of 68.23 ± 5.76 years were diagnosed with diabetes. There were 232 patients with both diabetes mellitus and heart failure. The patients in the diabetic heart failure group had significantly higher rates of asthma, COPD, PCI/CABG, prior myocardial infarction, and duration of diabetes mellitus than the patients in the diabetic group without heart failure. But there were no statistically significant variations in atrial fibrillation, hypertension, or smoking history (Table 1).

**Table 1:** Baseline characteristics of patients with type 2 diabetes according to the presence or absence of heart failure.

Characteristic	All patient (453)	HF (232)	Non-HF (221)	Odds ratio (95% CI)	*P* value
Age	68.23 ± 5.76	68.21 ± 7.98	70.23 ± 9.23	1.23 (0.78–1.56)	.054
Male-sex	265	134	131	1.05 (0.89–1.45)	.101
Median duration of diabetes	5.82 ± 0.87	6.12 ± 0.34	5.78 ± 0.76	0.89 (0.55–0.99)	.025
Current smoker	54	23	31	1.56 (1.24–1.98)	.142
Medical history					
Ischemic heart diasease	78	64	14	1.02 (0.76–1.48)	.011
Prior myocardial infarction	12	10	2	0.89 (0.80–1.11)	.004
Angina pectoris	35	32	3	0.97 (0.92–1.24)	.024
PCI/CABG	18	16	2	1.12 (0.99–1.35)	.038
Atrial fibrillation	7	6	1	0.88 (0.75–0.98)	.042
Hypertension	251	182	69	1.06 (0.96–1.39)	.081
TIA or stroke	48	41	7	1.24 (1.02–1.66)	.041
Asthma or COPD	89	48	41	1.28 (0.87–1.36)	.582
Medication					
Diuretics	125	68	57	1.03 (0.78–1.13)	.254
Mineralocorticoid antagonists	6	4	2	1.39 (1.18–1.42)	.088
ACE inhibitors or ARBs	235	151	84	1.02 (0.99–1.34)	.023
β-Blockers	115	85	30	0.79 (0.77–1.15)	.038
Lipid-lowering agents	285	185	100	0.89 (0.85–0.95)	.115
Oral antidiabetics	305	154	151	0.95 (0.91–1.25)	.259
Insulin	68	30	38	1.07 (0.95–1.34)	.337

ACE = angiotensin-converting enzyme, ARB = angiotensin II receptor blocker, CABG = coronary artery bypass grafting, CI = confidence interval, COPD = chronic obstructive pulmonary disease, HF = heart failure, PCI = percutaneous coronary intervention, TIA = transient ischemic attack.

When it comes to symptoms, the group with diabetes mellitus and heart failure had substantially more reports of swollen ankles or nocturia (*P* < .001), weariness or dyspnea (*P* < .001), and paroxysmal nocturnal dyspnea or orthopnea (*P* < .001). Nonetheless, there was no statistically significant difference between diabetics with heart failure and those without heart failure in terms of angina pectoris, palpitations, and claudication symptoms.

Upon physical examination, we also discovered that the group of diabetics with heart failure had substantially greater rates of wheezing or rhonchi (*P* = .021) and symptoms of water and salt retention (*P* = .008) than the group of diabetics without heart failure. Nonetheless, there were no statistically significant differences between diabetics with heart failure and those without heart failure in terms of systolic blood pressure (*P* = .358), diastolic blood pressure (*P* = .428), heart frequency (*P* = .114), body mass index (*P* = .089), laterally displaced or sustained apical impulse (*P* = .058), or systolic heart murmur (*P* = .481).

When compared to diabetics without heart failure, diabetics with heart failure had substantially greater levels of aberrant ST or T waves (*P* = .008), abnormal Q-waves (*P* = .008), and NT-proBNP ≥ 15 pmol/L (∼125 pg/mL) (*P* = .008). Table 2 shows that there were no significant differences between diabetics with heart failure and those without heart failure in terms of tachycardia, bradycardia, or pacemaker rhythm (*P* = .581), complete LBBB (*P* = .225), complete and incomplete RBBB (*P* = .214), LAHB (*P* = .369), LVH (*P* = .258), atrial fibrillation (*P* = .154), other ECG abnormalities (*P* = .208), mean creatinine level (*P* = .558), and HsCRP (*P* = .774).

**Table 2:** Symptoms and signs in 453 patients with type 2 diabetes.

Characteristic	All patient (453)	Non-HF (221)	HF (232)	Odds ratio (95% CI)	*P* value
Dyspnoea or fatigue	289	115	174	1.25 (0.89–1.68)	<.001
Paroxysmal nocturnal dyspnea or orthopnea	45	34	11	1.54 (1.05–1.98)	<.001
Reported swollen ankles or nocturia	295	178	117	1.05 (0.87–1.32)	<.001
Angina pectoris	32	18	14	0.89 (0.77–1.25)	.515
Palpitations	100	61	39	1.25 (0.58–1.36)	.121
Claudicational complaints	37	18	19	0.99 (0.89–1.62)	.311
Physical examination					
Systolic blood pressure (mm Hg)	159.25	146.11	162.84	0.97 (0.89–1.65)	.358
Diastolic blood pressure (mm Hg)	87.64	89.68	87.69	1.64 (1.08–2.69)	.428
Heart frequency (bpm)	71.26	71.58	69.58	1.22 (1.05–1.68)	.114
Body mass index (kg/m^2^)	28.69	29.74	27.66	1.54 (1.42–3.25)	.089
Wheezing or rhonchi	8.69	6.51	12.68	1.87 (0.98–2.61)	.021
Signs of water and salt retention	158	58	100	1.45 (0.77–2.15)	.008
Laterally displaced or sustained apical impulse	86	48	38	0.87 (0.77–0.95)	.058
Systolic heart murmur	110	56	54	1.48 (0.93–1.69)	.481
Additional tests					
Abnormal ECG	359	188	171	1.29 (0.94–1.64)	.217
Abnormal Q-waves	95	15	80	2.89 (1.48–3.62)	.001
ST or T waves abnormalities	114	42	72	1.14 (0.89–1.68)	.033
Tachycardia, bradycardia, or pacemaker rhythm	25	3	22	1.85 (0.99–1.98)	.581
Complete LBBB	36	8	28	2.61 (1.25–2.95)	.225
Complete and incomplete RBBB	42	6	36	2.15 (1.65–2.98)	.214
LAHB	38	9	29	1.24 (1.02–2.64)	.369
LVH	29	4	25	1.25 (0.58–2.66)	.258
Atrial fibrillation	18	2	16	0.98 (0.75–1.98)	.154
Other ECG abnormalities	154	60	94	1.68 (1.02–2.68)	.208
NT-proBNP ≥ 15 pmol/L (∼125 pg/mL)	142	34	108	2.69 (1.48–3.11)	<.001
Mean creatinin level (SD) in μmol/L	78.95 ± 18.52	79.36 ± 11.58	77.25 ± 15.64	1.22 (0.89–2.06)	.558
HsCRP (median, IQR) in mg/L	1.36 (0.88–2.61)	1.54 (1.05–3.65)	1.30 (0.69–1.85)	2.18 (1.58–3.02)	.774

CI = confidence interval, ECG = electrocardiogram, HF = heart failure, hsCRP = high-sensitivity C-reactive protein, IQR = interquartile range, LAHB = left anterior hemiblock, LBBB = left bundle branch block, LVH = left ventricular hypertrophy, NT-proBNP = N-terminal pro–B-type natriuretic peptide, RBBB = right bundle branch block, SD = standard deviation.

### 3.2. Univariate and multivariate Cox regression model

We examined fifteen variables in order to determine the factors linked to the risk of heart failure in individuals with diabetes. To investigate independent risk variables for HF, we conducted univariate and multivariate logistic regression analyses one after the other. The univariate analysis revealed that the development of diabetic heart failure (HF) was substantially correlated (*P* < .05) with aberrant ST or T waves, NT-proBNP, ischemic heart disease, atrial fibrillation, and diuretics. The development of diabetic heart disease (HF) was substantially correlated (*P* < .05) with ST or T wave abnormalities, NT-proBNP, ischemic heart disease, and atrial fibrillation, according to multivariate logistic regression analysis (Table 3).

**Table 3:** Univariate and multivariate Cox proportional hazards regression model.

	Univariate analysis			Multivariate analysis		
	HR	95% CI	*P* value	HR	95% CI	*P* value
Insulin	1.23	0.78 (0.67–1.42)	.234	1.11	1.23 (0.89–1.34)	.223
Median duration of diabetes	1.02	0.99 (0.98–1.23)	.076	0.77	0.89 (0.78–1.23)	.234
ST or T waves abnormalities	0.89	1.23 (1.22–2.34)	.023	0.23	0.72 (0.55–0.98)	.001
NT-proBNP	0.92	1.02 (0.89–1.13)	.012	0.89	1.11 (0.89–1.98)	.001
ischemic heart diasease	2.89	2.11 (1.87–2.90)	.023	1.23	0.88 (0.52–1.23)	.002
Atrial fibrillation	1.23	1.22 (0.78–2.34)	.021	0.78	1.23 (0.78–1.98)	.001
Diuretics	0.88	1.02 (0.98–1.77)	.003	0.98	1.89 (0.22–2.89)	.061

CI = confidence interval, HR = hazard ratio, NT-proBNP = N-terminal pro–B-type natriuretic peptide.

### 3.3. LASSO regression model

**Figure 1.**
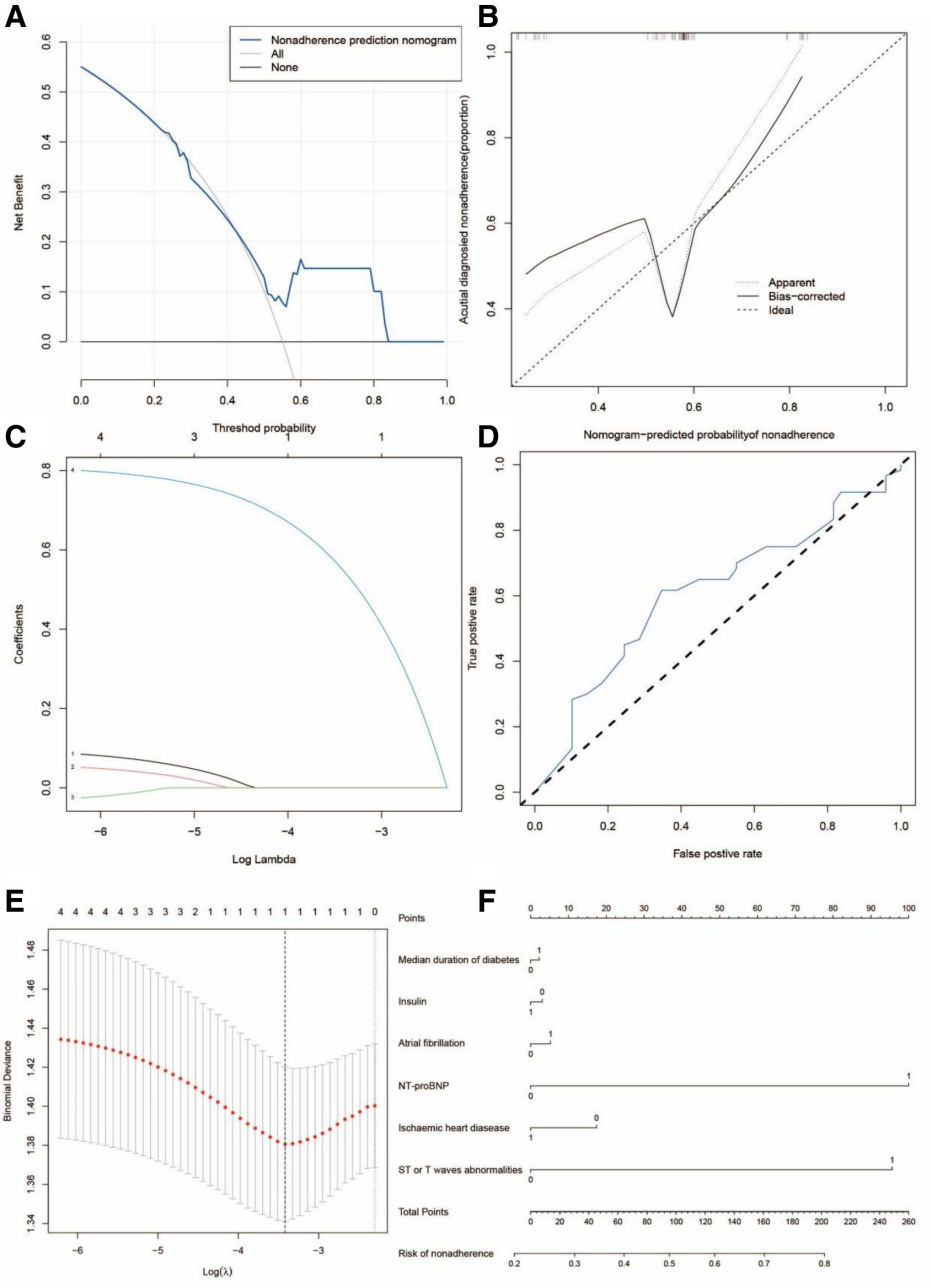
Development and validation of the prediction nomogram. (A) Decision curve analysis. (B) Calibration plot (apparent and bias-corrected vs ideal). (C) LASSO coefficient profiles. (D) ROC curve. (E) Cross-validation for optimal λ selection. (F) Final nomogram integrating median duration of diabetes, insulin use, atrial fibrillation, NT-proBNP, ischemic heart disease, and ST–T abnormalities. LASSO = least absolute shrinkage and selection operator, NT-proBNP = N-terminal pro–B-type natriuretic peptide, ROC = receiver operating characteristic.

In the LASSO regression model, 15 variables were narrowed down to 5 potential predictors with nonzero coefficients based on 453 patients in the cohort (Fig. 1A). Among them were insulin, the median length of diabetes, anomalies of the ST or T waves, NT-proBNP, ischemic heart disease, atrial fibrillation, and diuretics (Fig. 1B–D). The model including the previously indicated independent variables was used to construct the nomogram (Fig. 1E, F). On the other hand, high consistency was found in the calibration curve of the nonadherence risk nomogram for risk prediction in sepsis patients in this cohort. The model exhibited high discrimination, as shown by the concordance index of 0.984 (95% confidence interval, 0.252–1.369) for the prediction nomogram of the cohort and 0.958 following bootstrapping validation. The diabetic with heart failure-linked indicators nomogram’s decision curve analysis is shown in Figure 1C, and Figure 1E shows the AUC curve, which shows that the area under the curve was 0.964. To sum up, NT-proBNP might be a useful marker for the prognosis of diabetics who have heart failure.

## 4. Discussion

Heart failure risk is greatly increased by diabetes mellitus, a substantial risk factor that has been shown to accelerate the development of cardiovascular disease.^[12-15]^ In order to shed further light on the relationship between diabetes and heart failure, we examined the clinical results of individuals in our study^[10]^ who had both diabetes and heart failure. We used LASSO regression analysis and univariate and multivariate logistic regression analysis in our work to include NT-proBNP as a predictor of screening for diabetic heart failure.

Five machine learning algorithms were created and verified to forecast the likelihood of diabetic heart failure in patients based on these factors. The findings demonstrated that NT-proBNP and diuretics had the highest predictive potential for HF in patients with diabetes by evaluating the degree of calibration, discrimination, and clinical value of the univariate and multivariate logistic regression analysis and LASSO regression models.

Heart disease may occur as a result of prolonged hyperglycemia exposure.^[16]^ Advanced glycosylation end-products (AGEs) are glycated proteins or lipids that are produced after prolonged exposure to glucose. Previous studies have found that hyperglycemia causes the formation of AGEs.^[17]^ AGEs can cross-link with extracellular matrix proteins, increasing fibrosis and impairing myocardial diastole.^[18,19]^ AGEs can also cause intracellular damage by activating AGEs receptors, which increases cytosolic ROS and activates inflammatory pathways through NF-κB signaling.^[20,21]^ Patients with diabetes have chronic activation of the renin–angiotensin–aldosterone system.^[22]^ Increased afterload, vasoconstriction, and LV hypertrophy are all caused by elevated angiotensin II (AT-II) levels.^[23-25]^ AT-II also stimulates the synthesis of collagen, and the buildup of extracellular matrix proteins causes cardiac contractile dysfunction.^[26]^ Therefore, the increased damage linked to diabetic cardiomyopathy is a result of oxidative stress, inflammation, decreased mitochondrial energy, increased intracellular calcium handling, and neurohumoral activation.^[27-29]^

The following markers, which are very significant in diabetic heart failure, were present in our research. BNP comes first. As part of the natriuretic peptide family, BNP is related to atrial natriuretic peptide (ANP) and C-type natriuretic peptide (CNP).^[30]^ BNP binds to its A-type receptor to produce a range of effects, such as vasodilatation and inhibition of renin secretion.^[31]^ Individuals with diabetes have lower BNP concentrations, which may increase their risk of cardiovascular disease. Patients with diabetes and obesity also have higher plasma NT-pro-BNP levels, which may be linked to insulin resistance and long-term low-grade inflammation.^[31]^ Obese and diabetic patients also have elevated plasma NT-pro-BNP levels, which may be associated with chronic low-grade inflammation and insulin resistance.^[31]^ In older T2DM patients, NT-pro-BNP is a stronger independent predictor of short-term cardiovascular mortality than C-reactive protein and albumin excretion, according to a population-based study by Casale Monferrato.^[32]^ Dawson et al discovered that mild left ventricular anomalies and enhanced amplification of the ascending aortic pressure wave were associated with high-normal BNP levels.^[33]^ Diabetes and heart failure often deteriorate together, although the evidence of a correlation between the two was probably masked by the significant role natriuretic peptide plays in reducing insulin resistance, even in the metabolically hazardous state of heart failure.^[34]^ In an unselected AF patient sample, Hofer et al explored the independent associations between diabetes and HF and an elevated risk of CV death/HHF, finding that NT-proBNP enhanced risk assessment.^[35]^ The combination of NT-proBNP and other risk indicators may help identify diabetes patients who are at very high absolute risk.^[36]^ This suggests that long-term changes in NT-pro-BNP are more likely to represent the normal course of heart failure than to be a side effect of antidiabetic medication.^[37]^ Consistent with earlier findings, we also found that BNP is a significant biological marker in diabetes mellitus and heart failure.

Although congestion often occurs gradually prior to acute presentation, this book will refer to this situation as acute heart failure throughout the rest of it.^[38]^ Signs and symptoms of poor perfusion are only immediately evident in a small percentage of people with acute heart failure.^[39]^ Diuretics are a mainstay of heart failure treatment because of the critical role that congestion plays in the condition. According to guidelines, using loop diuretics is highly advised to reduce fluid excess symptoms.^[40]^ The relatively high percentage of diabetic patients with heart failure was another significant finding in this study. This raises questions about cardiologists’ awareness of the possibility of comorbid diabetes in their patients as well as potential challenges in diagnosing diabetes in people who may already be tired and experiencing thirst and frequency of urination due to diuretic therapy.

First, this was a retrospective study conducted at a single center, which may introduce selection bias and limit the generalizability of the findings. The patient population may not fully represent broader or more diverse clinical settings. Therefore, caution should be exercised when extrapolating the results to other populations. Second, although internal validation using bootstrapping demonstrated good model stability and discrimination, external validation was not performed. Without validation in independent cohorts from different institutions or regions, the robustness and transportability of the model remain uncertain. Future multicenter and prospective studies are needed to confirm its clinical applicability. Third, due to the retrospective nature of the study, some potentially relevant variables were not available or not consistently recorded in the medical records, such as medication adherence, socioeconomic status, lifestyle factors, and longitudinal glycemic control indicators. The omission of these variables may have affected the comprehensiveness of the model. Finally, the cross-sectional assessment of predictors and outcomes limits the ability to establish causal relationships. The model is intended for risk prediction rather than causal inference, and prospective longitudinal studies are warranted to better understand temporal relationships between predictors and heart failure development.

## 5. Conclusion

In summary, NT-proBNP was shown to be correlated, irrespective of other heart failure risk variables, with an increased incidence of incident heart failure in a large sample of individuals with type 2 diabetes. It’s yet unknown what processes underlie this relationship. Nevertheless, these results provide credence to the idea that preventing extreme hypoglycemia might lessen the chance of incident heart failure in those with type 2 diabetes. Preferential use of agents not linked to hypoglycemia might help accomplish this.

## Acknowledgments

We would like to thank all participants and our hospital.

## Author contributions

**Conceptualization:** Zhe Zhang, Dengao Li, Jumin Zhao, Huiting Ma, Fei Wang, Qinglian Hao.

**Data curation:** Zhe Zhang, Dengao Li, Jumin Zhao, Huiting Ma, Fei Wang, Qinglian Hao.

**Formal analysis:** Zhe Zhang, Dengao Li, Jumin Zhao, Huiting Ma, Fei Wang, Qinglian Hao.

**Funding acquisition:** Dengao Li.

**Investigation:** Dengao Li.

**Writing – original draft:** Dengao Li.

**Writing – review & editing:** Dengao Li.
